# Neutralizing Monoclonal Antibodies Reduce Human Cytomegalovirus Infection and Spread in Developing Placentas

**DOI:** 10.3390/vaccines7040135

**Published:** 2019-09-29

**Authors:** Takako Tabata, Matthew Petitt, June Fang-Hoover, Daniel C. Freed, Fengsheng Li, Zhiqiang An, Dai Wang, Tong-Ming Fu, Lenore Pereira

**Affiliations:** 1 Department of Cell and Tissue Biology, University of California, San Francisco, CA 94143, USA; takako.tabata@ucsf.edu (T.T.); petitt@mac.com (M.P.); hooverc109@aol.com (J.F.-H.); 2 Merck & Co., Inc., Kenilworth, NJ 07033, USA; dan_freed@merck.com (D.C.F.); fengsheng_li@merck.com (F.L.); dai_wang@merck.com (D.W.); tong-ming.fu@uth.tmc.edu (T.-M.F.); 3 Texas Therapeutics Institute, Brown Foundation Institute of Molecular Medicine, The University of Texas Health Science Center, Houston, TX 77030, USA; zhiqiang.an@uth.tmc.edu

**Keywords:** human cytomegalovirus, congenital infection, villus explants, cytotrophoblasts, placenta, transplacental transmission

## Abstract

Congenital human cytomegalovirus (HCMV) infection is a leading cause of birth defects worldwide, yet the most effective strategies for preventing virus transmission during pregnancy are unknown. We measured the efficacy of human monoclonal antibodies (mAbs) to HCMV attachment/entry factors glycoprotein B (gB) and the pentameric complex, gH/gL-pUL128–131, in preventing infection and spread of a clinical strain in primary placental cells and explants of developing anchoring villi. A total of 109 explants from five first-trimester placentas were cultured, and infection was analyzed in over 400 cell columns containing ~120,000 cytotrophoblasts (CTBs). mAbs to gB and gH/gL, 3-25 and 3-16, respectively, neutralized infection in stromal fibroblasts and trophoblast progenitor cells. mAbs to pUL128-131 of the pentameric complex, 1-103 and 2-18, neutralized infection of amniotic epithelial cells better than mAbs 3-25 and 3-16 and hyperimmune globulin. Select mAbs neutralized infection of cell column CTBs, with mAb 2-18 most effective, followed by mAb 3-25. Treatment of anchoring villi with mAbs postinfection reduced spread in CTBs and impaired formation of virion assembly compartments, with mAb 2-18 achieving better suppression at lower concentrations. These results predict that antibodies generated by HCMV vaccines or used for passive immunization have the potential to reduce transplacental transmission and congenital disease.

## 1. Introduction

Human cytomegalovirus (HCMV) is the most common virus transmitted in utero and is an infectious cause of birth defects. Congenital infection results in permanent neurological defects, mental retardation, hearing loss, visual impairment, and pregnancy complications, including intrauterine growth restriction (IUGR), preterm delivery, and stillbirth [1,2,3]. Primary maternal HCMV infection in the first trimester of pregnancy (9000/year in the U.S.) is associated with a 40% risk of transmission and results in the most severe damage [4]. Recurrent infections are significantly more common (30,000/year) [5] and are associated with a lower risk of transmission (0.2–2%) and better outcome but can still cause hearing deficiencies [6,7].

Given the major public health impact of congenital HCMV infection, the development of a vaccine capable of preventing transmission has long been a priority [8] (reviewed in [9,10,11]). A subunit vaccine based on the envelope glycoprotein B (gB), essential for infection of all cell types [12,13,14], reduced seroconversion by 50% in seronegative women, indicating partial protection [15] and a need for formulations that include other glycoproteins that elicit neutralizing antibodies [16,17,18]. HCMV pentameric complex gH/gL-pUL128–131, which promotes infection of epithelial and endothelial cells [19], has also been identified as a prominent target for neutralizing antibodies in cytotrophoblasts (CTBs) [20]. Importantly, a study in pregnant women with primary HCMV infection revealed associations between early development of pentamer-specific antibodies and the absence of transmission [21]. 

Although controversial, treatment of women who seroconvert during early pregnancy with hyperimmune globulin (HIG), a commercial preparation of antibodies from seropositive donors with high concentrations of HCMV-neutralizing antibodies [22], can significantly reduce transmission and sequelae in infected babies [23,24,25]. However, differences in recruitment of subjects based on time of seroconversion, HIG concentration, and frequency of treatment determine efficacy [26]. Women who seroconverted in the first trimester that were given more frequent and higher doses of HIG showed significant protection against HCMV transmission (7.5% versus 35.2%) [27]; this suggests that maintaining sufficiently high levels of antibodies confers protection. In principle, defined formulations containing the most effective antibodies at concentrations higher than occur in HIG could confer consistently better levels of protection. 

Our laboratory has studied HCMV infection using a model of anchoring villus explants that recapitulates development of human placentas in the first trimester of gestation [28,29]. To attach the placenta to the uterine wall, CTBs proliferate, breach the syncytiotrophoblast covering, and form cell columns [30,31]. At the distal tips of columns, differentiating CTBs invade the basal decidua and remodel uterine arterioles to form wide-bore, low-resistance blood vessels [32]. By mid-gestation, developing placental villi branch, increasing the surface of the syncytiotrophoblast bathed by maternal blood. In an ex vivo explant model of chorionic villus development, differentiating CTBs attach to and invade a Matrigel substrate, forming anchoring villi that are highly susceptible to HCMV infection. In subsequent studies of maternal immunity, we showed that placentas from the first trimester contain endogenous neutralizing IgG that generates immune complexes of virions that are phagocytosed by Hofbauer cells (fetal macrophages) in villus cores and largely prevents infection of CTB cell column in seropositive women [33]. 

Recently, a large panel of anti-HCMV human monoclonal antibodies (mAbs) from healthy seropositive donors was isolated and characterized for specificity, neutralizing capacity on epithelial cells, and binding affinities for whole virion and recombinant gB and pentameric complex [34]. Here we examined a select subset of these mAbs with high neutralizing activity targeting gB, gH/gL, and the pUL128–131 portion of the pentamer for their ability to neutralize infection in primary placental cells and explants of developing anchoring villi, as it is unknown which antibodies are most critical for preventing transplacental transmission. We also examined the ability of these mAbs to reduce virus spread in the tissue environment of villus explants when provided postinfection, modeling antibody treatment to reduce virus spread between cells in vivo. This study establishes a framework for evaluating mAbs with different specificities and properties of sera from recipients of HCMV vaccines for their potential efficacy in treatment and prevention of virus transmission during pregnancy.

## 2. Materials and Methods

### 2.1. HCMV Neutralizing Monoclonal and Control Antibodies

Human mAbs against HCMV gB (mAb 3–25, targeting site AD-2), gH/gL (mAb 3-16, gH-specific, targeting pentamer site 7) and the pUL128–131 portion of the pentameric complex (mAbs 1-103, targeting pentamer site 3, and 2–18, targeting pentamer site 1) were generated by Merck and Co., Inc. (Kenilworth, NJ, USA) [34]. Cytogam (HIG) (lot# 905322) was purchased from CSL Behring. Synagis, a human mAb to respiratory syncytial virus, was a gift from Trellis Bioscience, LLC (San Francisco, CA, USA) and was used as a negative control mAb.

### 2.2. Primary Cells and Human Placentas

Studies on HCMV infection of human placentas were approved by the Institutional Review Board of the University of California, San Francisco (UCSF). Placentas were obtained from uncomplicated deliveries at UCSF Moffitt–Long and Mission Bay Hospitals. Placentas from elective terminations were obtained from Advanced Bioscience Resources (Alameda, CA, USA). Amniotic epithelial cells (AmEpCs) were isolated from amniotic membranes dissected from placentas at 23.3 weeks and 38.6 weeks’ gestation. AmEpCs were cultured on fibronectin-coated 24-well plates in Dulbecco’s Modified Eagle Medium (DMEM)/F12 (Thermo Fisher Scientific, Walthan, MA, USA) supplemented with 20 ng/mL epidermal growth factor (EGF) (R&D Systems, Minneapolis, MN, USA), 10% fetal bovine serum (FBS), 1% non-essential amino acids, 55 µM 2-mercaptoethanol (Thermo Fisher Scientific), and antibiotics and antimycotics (UCSF Cell Culture Facility, San Francisco, CA, USA) as described previously [35]. Only first passage AmEpCs that were 100% positive by immunostaining for cytokeratin 19 (CK19) were used for experiments. Trophoblast progenitor cells (TBPCs) isolated from human chorionic membrane (15.6 weeks’ gestation), provided by O. Genbacev and S. Fisher [36], were cultured on gelatin-coated plates in DMEM/F12 (1:1) supplemented with 10 ng/mL basic fibroblast growth factor (bFGF) (R&D Systems), 10 µM SB431542 (Tocris Biosciences, Minneapolis, MN, USA) and 10% FBS. TBPCs 100% positive by immunostaining for cytokeratin with the rat monoclonal antibody 7D3 [37] and GATA4 were used up to passage 15 for experiments. Human placental fibroblasts (HPFs; 8 weeks’ gestation), a gift from D. Ilic [38], were grown in DMEM/M199 (4:1) with 1% amino acids and 10% FBS. Adult retinal pigment epithelial (ARPE-19) cells, highly differentiated cells, a gift from L. Hjelmeland [39], were grown in DMEM (Gibco) supplemented with 10% FBS as reported [40].

### 2.3. Virus Neutralization Assays

Stock virus from the clinical HCMV strain VR1814 was prepared as described previously [41]. HCMV neutralizing assays in cells were performed as reported [2,42,43]. Briefly, HPFs, TBPCs, and AmEpCs were seeded on cover slips in 24-well plates. The indicated dilutions of stock virus were preincubated with media alone (no antibody control) or with mAbs or HIG (Cytogam) at the indicated final concentrations for 1 h at 37 °C with moderate agitation. Virus multiplicity of infection (MOI) was adjusted to give a total of 600 to 800 infected cells/well by untreated virus. Virus-antibody mixtures were adsorbed to cells for 2 h in duplicate wells and washed. At 2 days postinfection (dpi), cells were fixed, permeabilized, and immunostained for HCMV immediate-early protein 1 (IE1). The number of infected cells was counted, and neutralizing titers were normalized to the no antibody control.

For assays in placental explants, chorionic villi were dissected from human placentas (8, 9, 11, and 14 weeks’ gestation) and 20 to 30 villus explants from each placenta were plated on Millicell-CM inserts (0.4 µm pore size, Millipore) coated with Matrigel in DMEM/F12 (1:1) with 10% FBS, penicillin/streptomycin and amino acids, conditions that enable differentiation of villus CTBs into invasive cells [44]. At 20 h after attachment to Matrigel, villus explants were infected with mixtures of VR1814 (3 × 10^6^ PFU) with mAbs, Cytogam, or Synagis overnight, washed and cultured two more days before fixation and frozen embedding. For quantification of infection in developing cell column CTBs, 1–3 sections of each explant were immunostained for cytokeratin 7 or 19 or by using anti-cytokeratin mAb 7D3, markers of CTBs, and HCMV IE1, and all cell columns were imaged on a Leica DMi8 microscope with a Leica DFC9000GT camera controlled by Leica Application Suite X software. Images were imported into Adobe Photoshop and cell columns identified by cytokeratin staining and morphology. Cell column nuclei (4’6-diamidino-2-phenylindole (DAPI) channel) were quantified in ImageJ using the particle analysis function, and HCMV IE1-positive nuclei were counted manually. The aggregate percentage of cell column CTBs infected in each condition from a placenta (1–2 explants per condition) was determined by combining numbers from all cell columns analyzed.

### 2.4. Virus Spread Inhibition Assays

HCMV spread inhibition assays were performed according to published methods [45] with modifications. For virus spread inhibition assays in developing placentas, explants of anchoring villi were prepared from a 10-week gestation placenta, cultured as described above, and infected 20 h after attachment to Matrigel with VR1814 (3 × 10^6^ PFU/explant). At 24 h postinfection, explants were washed and cultured with or without antibodies for 2 days before fixation and embedding. ARPE-19 cells plated in 24-well plates were infected with VR1814 at 1.5 or 2.5 PFU/cell. At 24 h, the culture media were removed, and cells were washed and cultured in media containing the indicated concentrations of antibodies up to 9 dpi. Quantification of infected cells was performed as above.

### 2.5. HCMV Titration

In virus spread inhibition experiments, titers of VR1814 released into conditioned media (CM) and intracellular titers were measured by rapid immunofluorescence-based infectivity assays in ARPE-19 cells, as previously described [35]. For measurement of intracellular titers, cells were collected, resuspended in media, and lysed by 4 freeze–thaw cycles.

### 2.6. Antibodies and Reagents

The following antibodies were purchased: mouse mAbs to HCMV IE (MilliporeSigma, Burlington, MA, USA), CD68 and CK7 (Dako, Carpinteria, CA, USA), and CD32A (Abcam, Cambridge, UK); a rabbit mAb to CD163 (Novus Biologicals, Centennial, CO, USA); and a rabbit polyclonal antibody to human cytokeratin 19 (ProteinTech, Rosemount, IL, USA). We also used mAbs CH433 (anti-IE1 (UL123), immediate-early protein), CH112-2 (anti-gB (UL55), early protein), CH19 (anti-pp28 (UL99), late protein) [46], and M23 (anti-UL112/UL113, early proteins) [47]. Rabbit polyclonal antibody to the human neonatal Fc receptor (FcRn) for IgG was a gift from Neil Simister [48]. Rat anti-human cytokeratin mAb (clone 7D3) was a gift from S. Fisher [37], and guinea pig anti-HCMV gB was a gift from R.L. Burke (Chiron Corporation, Emeryville, CA, USA). To quantify apoptosis, we used the In Situ Cell Death Detection Kit, Fluorescence (MilliporeSigma, Burlington, MA, USA) according to the manufacturer’s protocol following IE1 staining.

### 2.7. Immunofluorescence and Imaging

Cells grown on cover slips were fixed with 4% paraformaldehyde and permeabilized with 0.1% Triton X-100. Frozen tissues were cut into 5 µm sections. For double and triple immunostaining, after blocking with 3–5% normal serum matching the secondary antibody source for cells and bovine serum albumin plus 3–5% normal serum for explants, cells or tissue sections were simultaneously incubated with primary antibodies from different species followed by incubation with secondary antibodies labeled with fluorescein isothiocyanate (FITC), rhodamine red-X (RRX), or Cy5 (Jackson ImmunoResearch, West Grove, PA, USA). Nuclei were stained with DAPI (Vector Laboratories, Burlingame, CA, USA). Alternatively, cells and tissues were incubated with primary antibodies against cellular proteins overnight, followed by incubation with secondary antibodies, then stained with antibodies to HCMV proteins. Images were obtained using a Leica DMi8 microscope equipped with a Leica DFC9000GT camera controlled by Leica Application Suite X software. Images of whole explants were taken on a Leica M125 stereomicroscope equipped with a Leica MC170HD camera.

## 3. Results

### 3.1. mAbs Have Potent HCMV Neutralizing Activities in Primary Placental Cells

#### 3.1.1. Human Placental Fibroblast (HPF) Infection Is Blocked by mAbs to gB and gH/gL

**Figure 1:**
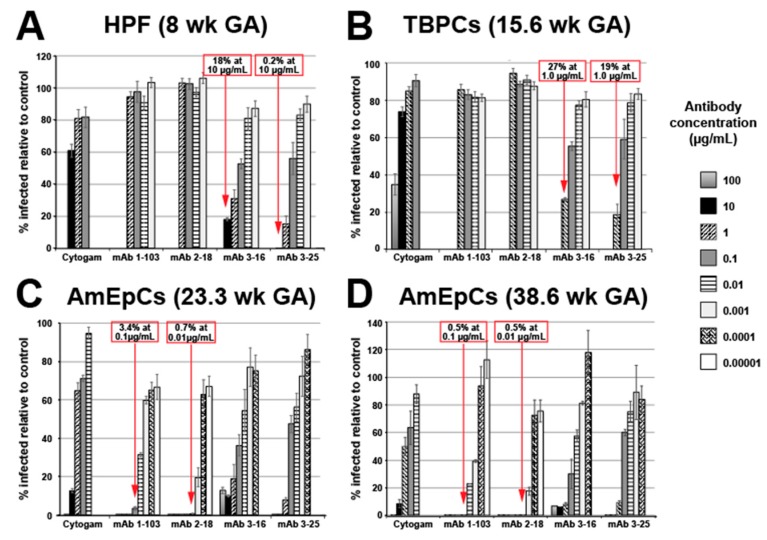
Human monoclonal antibody (mAb) neutralization of VR1814 infection of primary human placental cells. Primary placental cells were infected with VR1814 preincubated with medium alone (no antibody control) or medium containing antibodies. Cells were fixed at 2 days postinfection and immunostained for human cytomegalovirus (HCMV) immediate-early protein 1 (IE1), and IE1+ cells were counted. Counts were normalized to the no antibody control and are expressed as percentages (mean ± SE). (**A**) Results from four independent experiments (*n* = 2–8) on human placental fibroblasts (HPFs) from an 8-week gestation placenta. (**B**) Results from four independent experiments (*n* = 8) on trophoblast progenitor cells (TBPCs) from a 15.6-week gestation placenta. (**C**) Results from three independent experiments (*n* = 2–6) on amniotic epithelial cells (AmEpCs) from a 23.3-week gestation placenta. (**D**) Results from two independent experiments (*n* = 2–4) on AmEpCs from a 38.6-week gestation placenta. Red boxes and arrows highlight the most potent neutralizing activities. *n* = total replicates counted across all experiments. GA, gestational age.

To evaluate the relative efficacies of anti-HCMV mAbs of different specificities and Cytogam in neutralization of infection of placental cells, we measured the neutralizing activities of mAbs against pUL128-131 of the pentameric complex (mAbs 1-103 and 2-18), gH/gL (mAb 3–16), and gB (mAb 3-25) and Cytogam on primary placental cells using the pathogenic clinical HCMV strain VR1814. In HPFs, anti-gB and anti-gH/gL mAbs neutralized VR1814 with moderate potency, achieving 50% neutralization at ~1.4 µg/mL, whereas anti-pentamer mAbs failed to prevent virus entry into HPFs at the concentrations tested (Figure 1A). HIG had a neutralizing effect at the highest concentration tested (10 µg/mL, ~40% inhibition).

#### 3.1.2. Trophoblast Progenitor Cell (TBPC) Infection Is Blocked by mAbs to gB and gH/gL

We reported that HCMV replicates in multipotent TBPCs—precursors of the mature placental cell types, syncytiotrophoblasts and CTBs [49]. TBPCs are fully permissive for HCMV infection, and viral entry is independent of the pentameric complex, based on infection by a UL131A deletion mutant and the finding that anti-gB mAb TRL345 neutralizes infection ~100-fold more potently than HIG [43]. In agreement with our previous findings, the anti-gB mAb 3-25 efficiently blocked virus entry into TBPCs (Figure 1B). mAb 3-16 (anti-gH/gL) also reduced infection of TBPCs (Figure 1B), and the neutralizing activities of mAbs 3-25 and 3-16 were similar to their activities in HPFs. In contrast, anti-pentamer antibodies (mAbs 1-103 and 2-18) had little or no neutralizing activity at the concentrations tested (0.001–1.0 µg/mL). Cytogam partially blocked virus entry (~66% inhibition) at the highest concentration tested (100 µg/mL).

#### 3.1.3. Amniotic Epithelial Cell (AmEpC) Infection Is Strongly Inhibited by Anti-Pentamer mAbs

Primary AmEpCs from amniochorionic membranes are self-renewing with stem cell characteristics and support persistent HCMV infection [35]. We carried out neutralizing assays with VR1814 using AmEpCs of mid- and late-gestation placentas. In agreement with our previous studies [35], anti-pentamer mAbs potently neutralized infection. mAb 2–18 exhibited the greatest activity, reducing infection by ~99% at 0.01 µg/mL, followed by mAb 1–103, with an approximately 10-fold lower potency (Figure 1C,D). The next most potent were mAbs 3-16 and 3-25, having IC50 values 50–100-fold (mAb 3-16) and 6–40-fold (mAb 3-25) lower than that of Cytogam, although requiring 100- to 1000-fold higher concentrations of antibodies than did mAb 2-18 to achieve similar levels of neutralization. Taken together, our studies showed that mAbs to HCMV glycoproteins could prevent infection of diverse placental cell types at different concentrations.

### 3.2. mAbs Specific to HCMV Proteins Neutralize Infection of Cell Column CTBs in Anchoring Villus Explants

**Figure 2:**
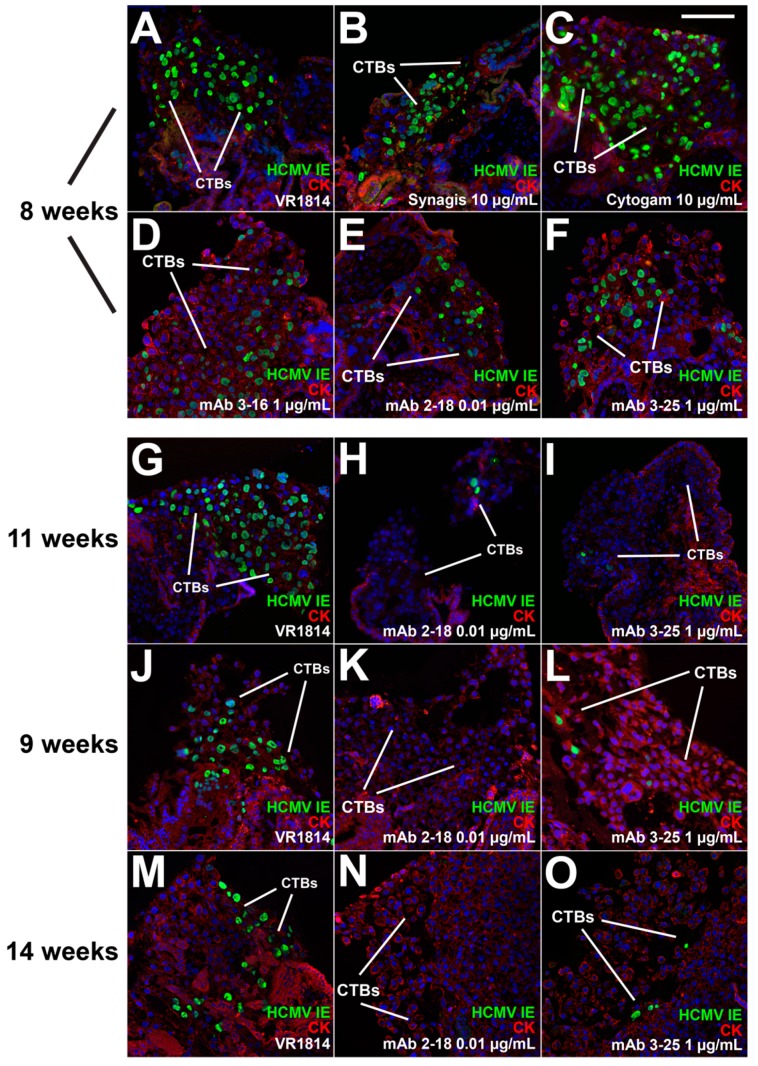
Neutralization of HCMV infection of cell column cytotrophoblasts (CTBs) in anchoring villus explants. Immunofluorescence staining for HCMV IE1 and cytokeratin (CK) (7D3) in sections of anchoring villus explants from four placentas of different gestational ages infected with VR1814 alone or mixed with antibodies (i.e., immune complexes) at the indicated concentrations. In two experiments (8 and 11 weeks’ gestation), single concentrations of mAbs were compared to single concentrations of Cytogam and the negative control antibody Synagis. In two experiments (9 and 14 weeks), multiple concentrations of mAbs 2-18, 3-16, and 3-25 were compared. Representative images of all conditions are shown for the 8-week placenta (**A**–**F**), whereas only results for VR1814 alone and VR1814 mixed with mAbs 2-18 and 3-25 are shown for explants from the 11-, 9-, and 14-week gestation placentas. (**G**–**O**) Quantitative results for all experimental conditions are shown in Figure 3. (**A**–**F**) Explants from an 8-week gestation placenta showing infection with VR1814 alone (**A**) and infection with VR1814 mixed with negative control antibody Synagis (10 µg/mL; **B**), Cytogam (10 µg/mL; **C**), mAb 3-16 (1.0 µg/mL; **D**), mAb 2-18 (0.01 µg/mL; **E**), and mAb 3-25 (1.0 µg/mL; **F**). (**G**–**O**) Selected images from neutralization studies in explants from three placentas showing infection alone (**G**,**J**,**M**) and infection with virus mixed with either mAb 2-18 (0.01 µg/mL; **H**,**K**,**N**) or mAb 3-25 (**I**,**L**,**O**). Scale bar, panel C = 100 µm. Nuclei were stained with DAPI.

**Figure 3:**
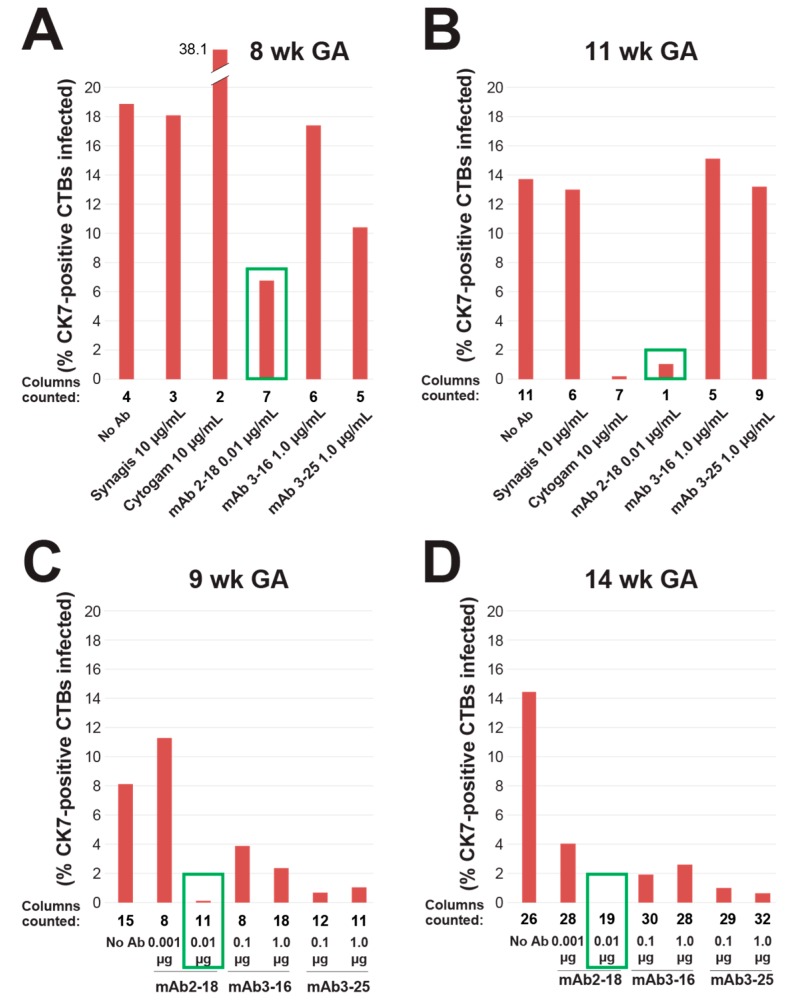
mAbs specific to gB, gH/gL, and the pentamer complex neutralize infection of CTB cell columns. Quantification of antibody neutralizing activities on anchoring villus explants from four placentas of 8 (**A**), 11 (**B**), 9 (**C**), and 14 weeks (**D**) gestational age (GA) corresponding to explants shown in Figure 2. Explants were infected with VR1814 preincubated with medium alone or with antibodies (i.e., immune complexes) at the concentrations indicated, and 341 cell columns were analyzed. Bars indicate aggregate percentage of cell column CTBs infected (HCMV IE1+) among all cell columns analyzed. Numbers of cell columns counted is indicated below each bar. Green boxes highlight the most potent neutralizing antibody.

Under the culture conditions used for explants, CTBs differentiate and invade the Matrigel substrate prior to infection of anchoring villi. We reported that VR1814 replicates in differentiating CTBs in proximal cell columns and reduces outgrowth [44]. To assess the therapeutic potential of anti-HCMV mAbs in the tissue environment of developing placentas, we performed neutralizing assays with mAbs 2-18, 3-16, and 3-25, Cytogam, and control mAb Synagis on anchoring villus explants from four first and early second trimester placentas. VR1814 was mixed with antibodies and used for infection, after which explants were washed and cultured in virus- and antibody-free medium. Explants were fixed at 3 dpi, and fixed-frozen sections were immunostained for HCMV IE1 and a CTB marker (Figure 2). Total and infected CTBs were counted in 341 cell columns, and the aggregate percentage of infected cells was determined for each condition (Figure 3).

In experiments in villus explants from the 8- and 11-week gestation placentas infected with VR1814 alone or mixed with antibody, we compared single concentrations of each mAb, Cytogam, and control mAb Synagis. In the 8-week gestation explants, mAb 2-18 exhibited the greatest neutralizing activity at 0.01 µg/mL, followed by mAbs 3-16 and 3-25 at 1 µg/mL (Figure 2A–F, Figure 3A). A 1000-fold higher concentration of Cytogam (10 µg/mL) and control antibody Synagis (10 µg/mL) each had no neutralizing effect. In explants from the 11-week gestation placenta, only mAb 2-18 (0.01 µg/mL) had a strong inhibitory effect, while mAbs 3-16 and 3-25 and Synagis had no significant neutralizing activity, even though tested at higher concentrations (Figure 2G–I, Figure 3B). Cytogam at 100 µg/mL, a 10,000-fold greater concentration than that of mAb 2-18, also neutralized infection.

In two experiments on anchoring villus explants from placentas of 9 and 14 weeks’ gestation, we compared two concentrations of mAbs 2-18, 3-16, and 3-25. In both sets of explants, all mAbs reduced infection at the concentrations tested, but mAb 2-18 achieved the greatest neutralization at the lowest concentration (0.01 µg/mL) (Figure 2J–O, Figure 3C,D). These results indicate that comparatively very low concentrations of highly neutralizing mAbs directed against pUL128-131 of the pentamer (1000–10,000-fold lower than Cytogam) block infection of differentiating CTBs. Moreover, mAbs targeting gB and gH/gL were also more effective than Cytogam at lower concentrations and could enhance overall protection by blocking infection of additional cell types at the uterine-placental interface.

### 3.3. Hofbauer Cells in Villus Cores Phagocytose IgG-Virion Complexes

**Figure 4:**
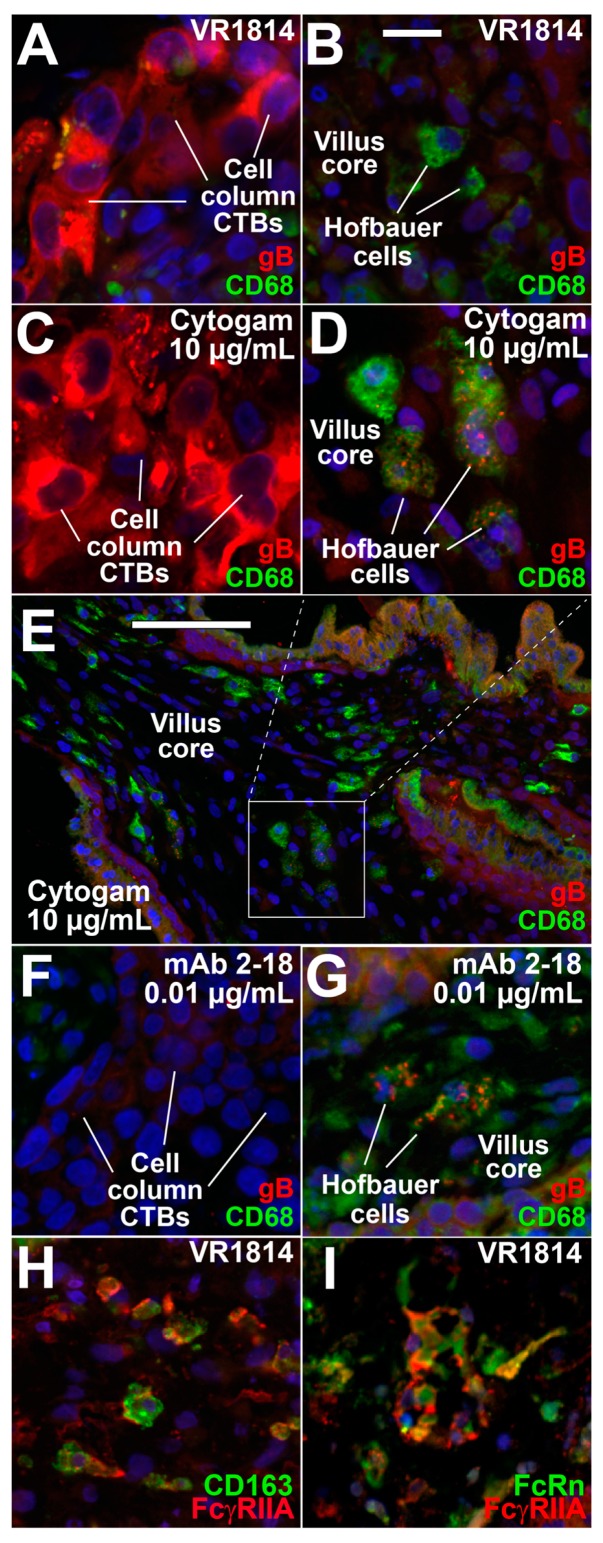
Hofbauer cells in villus cores phagocytose IgG-virion immune complexes transcytosed by syncytiotrophoblasts in anchoring villus explants. (**A**–**G**) Immunofluorescence staining for HCMV gB and CD68, a marker of Hofbauer cells, in sections of anchoring villus explants from an 8-week gestation placenta infected with VR1814 alone or mixed with antibodies (immune complexes). Cell column CTBs (**A**) and Hofbauer cells in the corresponding villus core (**B**) infected with VR1814 alone. Cell column CTBs (**C**) and Hofbauer cells in the corresponding villus core (**D**,**E**) infected with VR1814 mixed with 10 µg/mL Cytogam. Cell column CTBs (**F**) and Hofbauer cells in the corresponding villus core (**G**) infected with VR1814 mixed with 0.01 µg/mL mAb 2-18 (yellow frame). Immunofluorescence staining of villus explants from a 14-week gestation placenta for FcγRIIA and Hofbauer cell marker CD163 (**H**) and for FcγRIIA and FcRn (**I**). Scale bar for panels (**A**–**D**) and (**F**–**I**) in panel B = 20 µm. Scale bar, panel E = 100 µm. Nuclei were stained with DAPI. Similar results were seen in explants from 11 weeks’ gestation (data not shown).

We reported that Hofbauer cells in first-trimester placentas from seropositive donors with HCMV neutralizing antibodies contain IgG and gB colocalized in cytoplasmic vesicles without HCMV IE1 protein in nuclei, indicating phagocytosis of virion-immune complexes without infection following transcytosis by the neonatal Fc receptor (FcRn) in syncytiotrophoblasts [33]. To determine whether defined IgG-virion complexes would be cleared similarly, villus explants from the 8 and 11-week gestation placentas infected with VR1814 alone or with antibody mixtures were immunostained for HCMV gB and CD68, a macrophage marker. Infection with VR1814 alone showed strong gB staining in CTB cell columns (Figure 4A), but no gB was found in Hofbauer cells in villus cores (Figure 4B). Strong cytoplasmic gB was also detected in cell column CTBs of explants infected with VR1814 pretreated with 10 µg/mL Cytogam (Figure 4C), indicating infection; however, the Hofbauer cells in villus cores contained gB in cytoplasmic vesicles, in accord with phagocytosis of IgG-virion complexes (Figure 4D,E). Explants infected with VR1814 pretreated with 0.01 µg/mL mAb 2-18 showed few CTBs expressing gB in cell columns (Figure 4F), whereas Hofbauer cells contained gB in cytoplasmic vesicles (Figure 4G), indicating phagocytosis of IgG-virion complexes. mAb 2-18 enabled uptake of immune complexes by Hofbauer cells at a 1000-fold lower concentration than did Cytogam, suggesting it strongly promotes virion clearance. gB uptake was detected in only 1.3 ± 0.7% of Hofbauer cells in villi infected with VR1814 alone. In contrast, gB uptake was significantly higher in explants pretreated with 10 µg/mL Cytogam (48.8 ± 12.6%, *p* < 0.01) and 0.01 µg/mL mAb 2-18 (32.4 ± 5.6%, *p* < 0.0001). gB uptake was also observed in explants treated with 1 µg/mL mAb 3-16 (13.0 ± 4.3%, *p* < 0.05) and 3-25 (25.1 ± 7.1%, *p* < 0.01). To identify potential Fc receptors (FcRs) for IgG on Hofbauer cells that could mediate antibody-dependent phagocytosis, we costained explants for FcγRIIA (CD32A), an activating FcR, CD163, macrophage marker, and FcRn that binds IgG at low pH. A large percentage of CD163-positive cells also expressed FcγRIIA, and a subset of FcγRIIA-expressing cells also expressed FcRn (Figure 4H,I). Together the results show that Hofbauer cells in villus cores phagocytose IgG-virion complexes irrespective of CTB infection and suggest that FcγRIIA and FcRn cooperate to mediate phagocytosis and intracellular trafficking of immune complexes in the endocytic pathway.

### 3.4. Antibody Inhibition of Cell–Cell Spread of HCMV and Apoptosis in CTB Cell Columns in Anchoring Villus Explants

**Figure 5:**
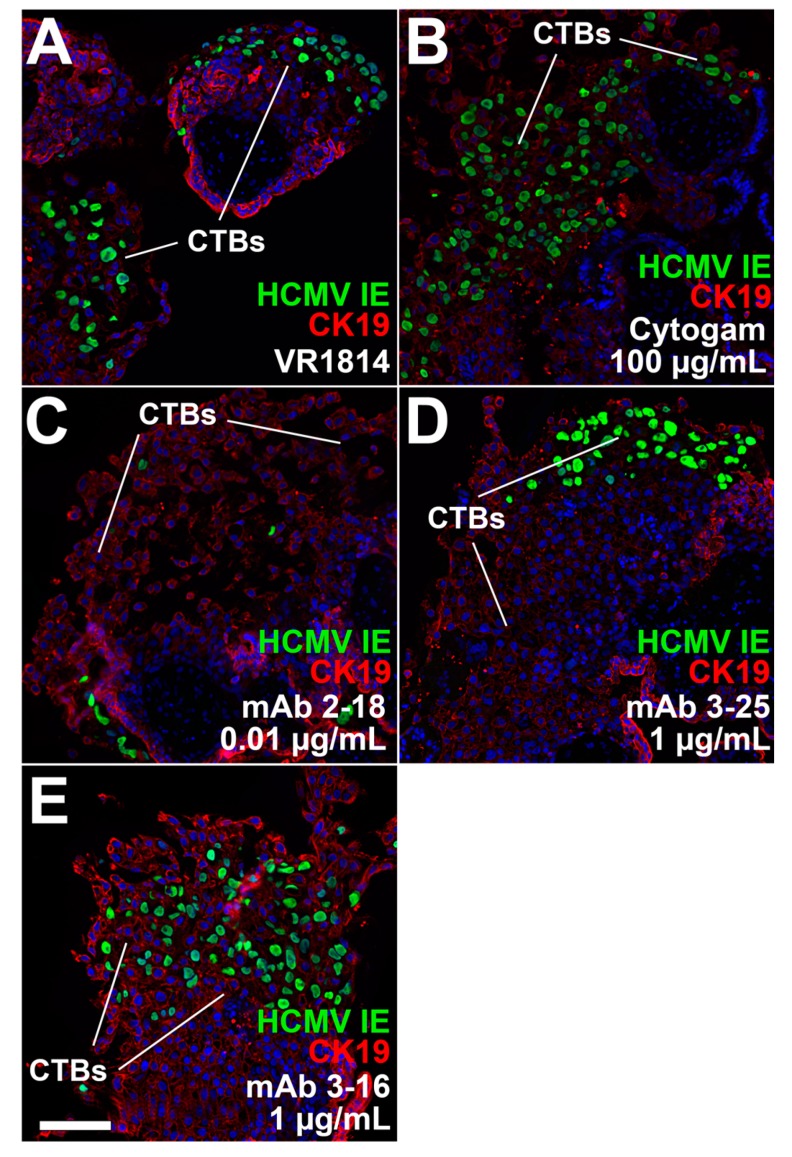
Anti-pentamer mAb 2-18 treatment postinfection reduces cell-cell virus spread in CTB cell columns. Immunofluorescence staining for HCMV IE proteins and cytokeratin (CK) 19 in sections of villus explants from a 10-week gestation placenta infected with VR1814 and treated postinfection with antibodies for two days. (**A**) Untreated VR1814-infected explant. (**B**) Explants treated with 100 µg/mL Cytogam, (**C**) 0.01 µg/mL mAb 2-18, (**D**) 1.0 µg/mL mAb 3-25, and (**E**) 1.0 µg/mL mAb 3-16. Scale bar, panel E = 100 µm. Nuclei were stained with DAPI.

**Figure 6:**
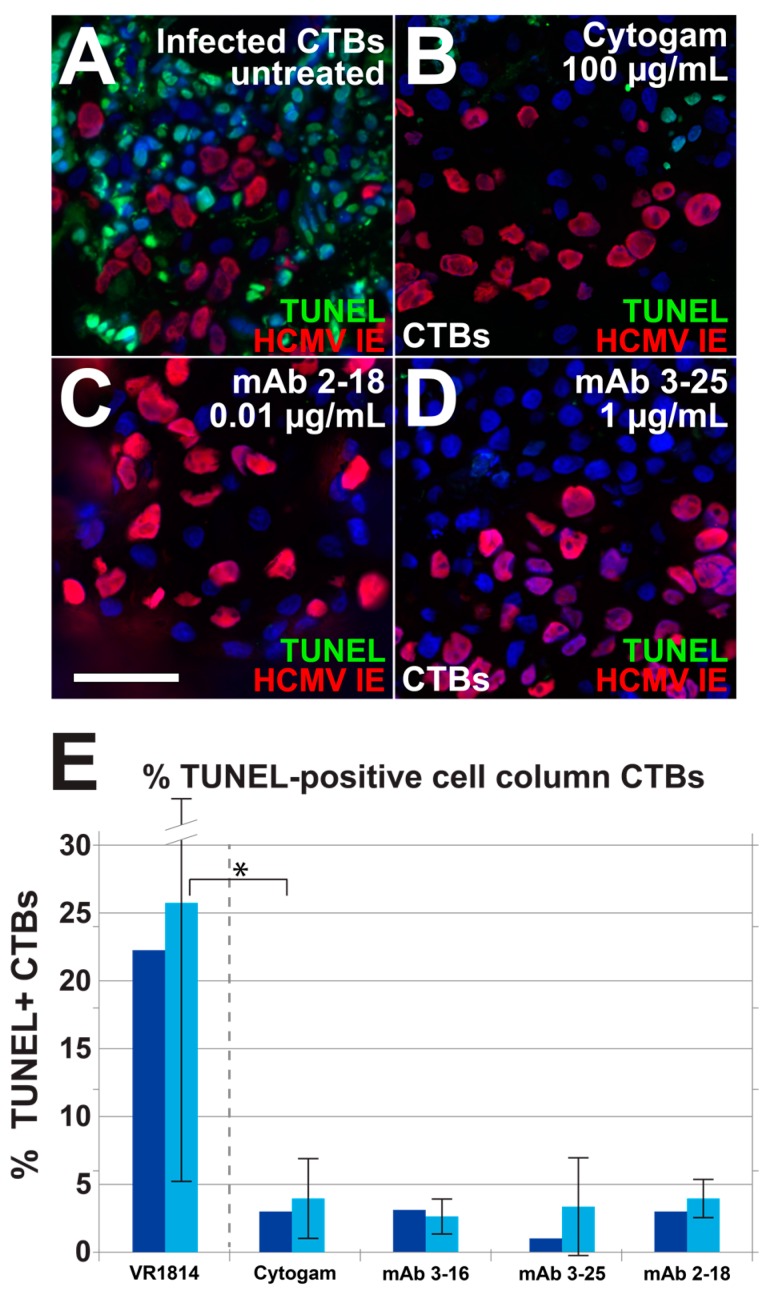
Antibody treatment of VR1814-infected anchoring villus explants postinfection reduces apoptosis in CTB cell columns. Terminal deoxynucleotidyl transferase dUTP nick end labeling (TUNEL) assay and immunostaining for HCMV IE1 in sections of anchoring villi described in Figure 5. (**A**) Control untreated explant. (**B**) Explants treated with 100 µg/mL Cytogam, (**C**) 0.01 µg/mL mAb 2-18, and (**D**) 1.0 µg/mL mAb 3-25. Scale bar, panel C = 50 µm. Nuclei were stained with DAPI. (**E**) Graph showing the aggregate percentages of TUNEL-positive cell column CTBs (dark blue bars) and means of the percentages of TUNEL-positive CTBs in individual cell columns (light blue bars), along with standard deviations. * *p* < 0.05.

Effective antibody-based protection against transmission in pregnancy may require antibodies that reduce cell-cell spread of HCMV in developing placentas and apoptosis associated with pathology [2,43,49]. We therefore tested the ability of mAbs 2-18, 3-16, and 3-25 to limit cell-cell spread in developing placentas when added postinfection. One day after infection with VR1814, anchoring villus explants from a 10-week gestation placenta were treated with mAbs at concentrations shown to be effective in neutralization experiments or left untreated and analyzed two days later. Immunostaining for HCMV IE1 showed significant numbers of infected CTBs in cell columns in untreated explants (Figure 5A). Treatment with Cytogam (100 µg/mL) had little effect on infection (Figure 5B), but mAb 2-18 at a lower concentration (0.01 µg/mL) reduced infection (Figure 5C). By contrast, little or no reduction of infection was observed in explants treated with mAbs 3-16 and 3-25 at higher concentrations (1.0 µg/mL; Figure 5D,E). HCMV infection in primary CTBs led to the rapid loss of bystander cells mediated by HCMV IE1-induced tumor necrosis factor (TNF)-α secretion [50]. We used terminal deoxynucleotidyl transferase dUTP nick end labeling (TUNEL) assay to determine if decreased IE expression in antibody-treated explants reduces apoptosis in bystander cells. Without antibody treatment, infected CTBs in explants were surrounded by uninfected apoptotic cells (Figure 6A), whereas explants treated with antibodies showed reduced numbers of apoptotic cells (Figure 6B–E). 

**Figure 7:**
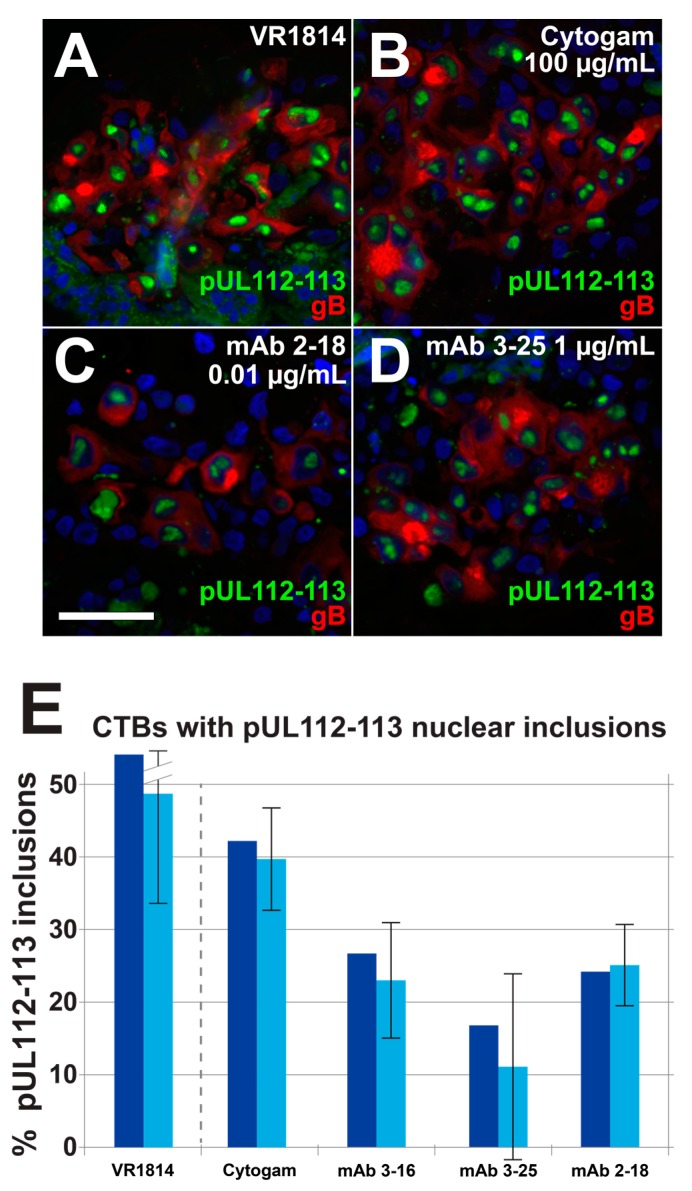
Antibody treatment reduces gB expression and pUL112-113 in nuclear inclusions. Immunofluorescence staining for HCMV pUL112-113 and gB in sections of anchoring villi described in Figure 5. (**A**) Control untreated explant. (**B**) Explants treated with 100 µg/mL Cytogam, (**C**) 0.01 µg/mL mAb 2-18, and (**D**) 1.0 µg/mL mAb 3-25. (**E**) Percentages of gB-expressing cells with pUL112-113 nuclear inclusions versus punctate signal, including both aggregate percentages (dark blue bars) and means of the percentages in individual cell columns (light blue bars). Scale bar, panel C = 50 µm. Nuclei were stained with DAPI.

**Figure 8:**
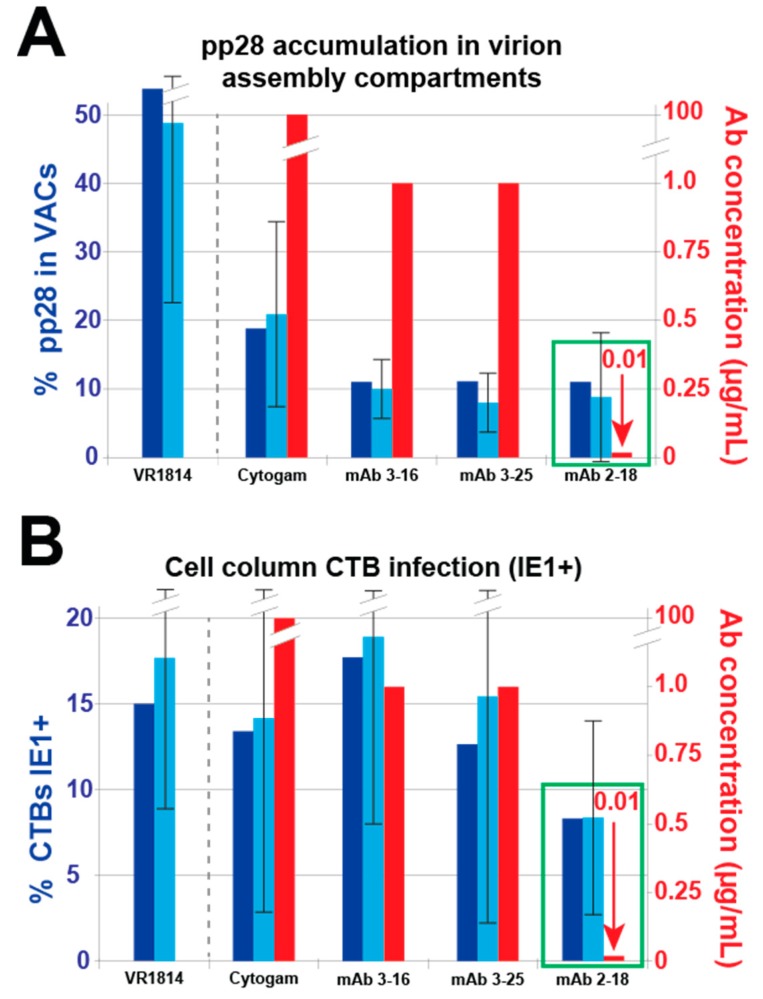
Antibody treatment reduces pp28 in viral assembly compartments (VACs) and cell–cell spread in infected anchoring villi. (**A**) Quantification of pp28 in VACs of infected cell column CTBs in explants treated with antibodies postinfection (described in Figure 5). The percentage of gB-positive cells showing pp28 immunostaining in VACs was determined (left axis, blue), including both aggregate percentage of CTBs with pp28 in VACs (dark blue bars) and the mean of percentages from individual cell columns (light blue bars), along with standard deviation, and compared with antibody concentration (right axis, red). A total of 28 cell columns were analyzed. (**B**) Aggregate percentages (dark blue bars) and mean percentages (light blue bars) with standard deviations of IE1-positive cell column CTBs in 10-week gestation explants treated with antibodies postinfection, based on quantification of infection in 80 cell columns (left axis, blue) compared with antibody concentration (right axis, red). Green boxes highlight the antibody with the most potent effects on pp28 and cell–cell spread.

To determine whether antibody treatment of anchoring villus explants postinfection alters HCMV replication kinetics in infected cell column CTBs, we examined expression of gB, initially made at low levels but strongly upregulated late in infection [51]; we also examined early proteins pUL112-113, which accumulate at sites of DNA replication, with the highest levels in large nuclear inclusions at late times [47,52], and directly interact with IE2 and DNA polymerase accessory protein pUL44 [53]. Immunostaining showed reduced levels of gB in explants treated with mAbs 2-18 and 3-25 relative to untreated explants, but little or no effect by either mAb 3-16 or Cytogam (Figure 7). Similarly, in explants treated with mAbs, there were decreased numbers of pUL112-113 nuclear inclusions. These results suggest suppressed viral DNA replication, which leads to reduced expression of late viral gene products [51]. We next determined the total number of gB-expressing cells and the number also exhibiting strong pp28 localization to viral assembly compartments (VACs) in 28 cell columns from control infected explants and those treated with antibodies postinfection (Figure 8A). While all three mAbs and Cytogam reduced the percentages of gB-expressing cells with pp28 in VACs, mAb 2-18 achieved reduction similar to those of mAbs 3-16 and 3-25 at a 100-fold lower concentration and a greater reduction than Cytogam at a 10,000-fold lower concentration. To compare the effects of different antibodies on cell-cell spread of virus within cell columns, we quantified infection in a total of 80 cell columns in sections immunostained for HCMV IE1 and CK19 and found that a low concentration of mAb 2-18 (0.01 µg/mL) reduced infection relative to untreated controls, whereas the higher concentrations of mAbs 3-16, 3-25, and Cytogam had little to no effect (Figure 8B). Thus, antibody treatment postinfection reduces or delays HCMV replication and can limit cell-cell spread in anchoring villi of placentas, with anti-pUL128-131 of the pentameric complex mAb 2-18 having the most potent effects.

### 3.5. Neutralizing Antibodies Inhibit Cell–Cell Spread of HCMV in ARPE-19 Cells

**Figure 9:**
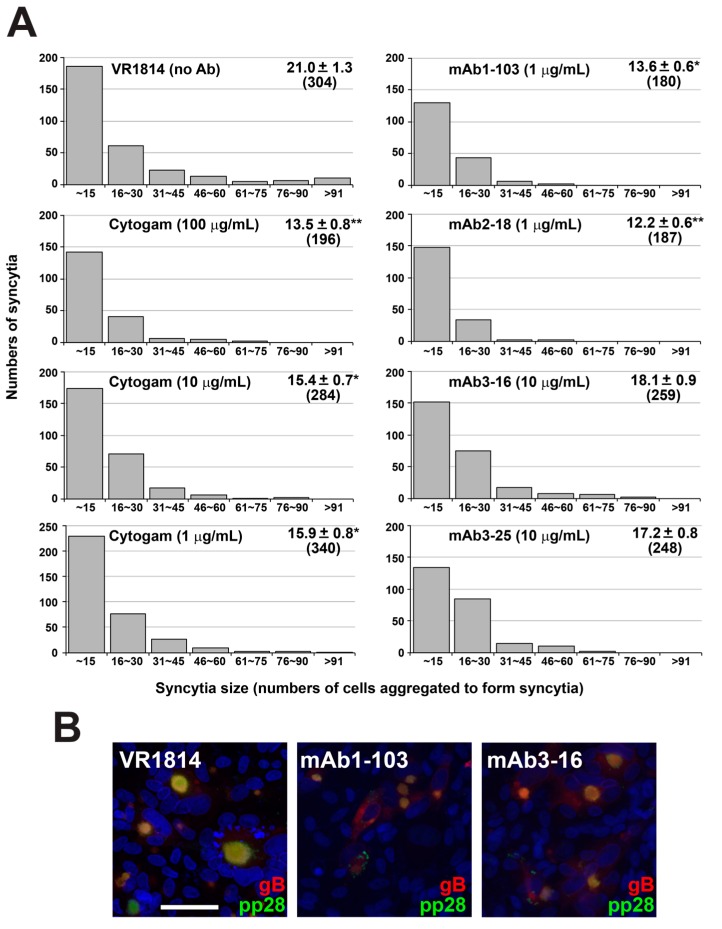
Antibody treatment postinfection reduces syncytium formation in infected adult retinal pigment epithelial (ARPE-19) cells. ARPE-19 cells were infected with VR1814 (2.5 PFU/cell) and treated postinfection with medium alone or with antibodies. (**A**) Representative histograms showing syncytia size (numbers of cells aggregated to form syncytia) from two experiments. For each condition, 14 fields of 20× images were counted. Numbers indicate the mean syncytia size ± SE and total number of syncytia formed (indicated in parentheses). *p*-Values based on Mann–Whitney comparisons between VR1814 infection alone and treatment of antibodies are shown (* *p* < 0.05, ** *p* < 0.01). (**B**) Immunofluorescence staining of gB and pp28 in ARPE-19 cells infected with VR1814 (2.5 PFU/cell) and treated postinfection with medium alone, mAb 1-103 (1 µg/mL) and mAb 3-16 (10 µg/mL). Scale bar = 100 µm. Nuclei were stained with DAPI.

**Figure 10:**
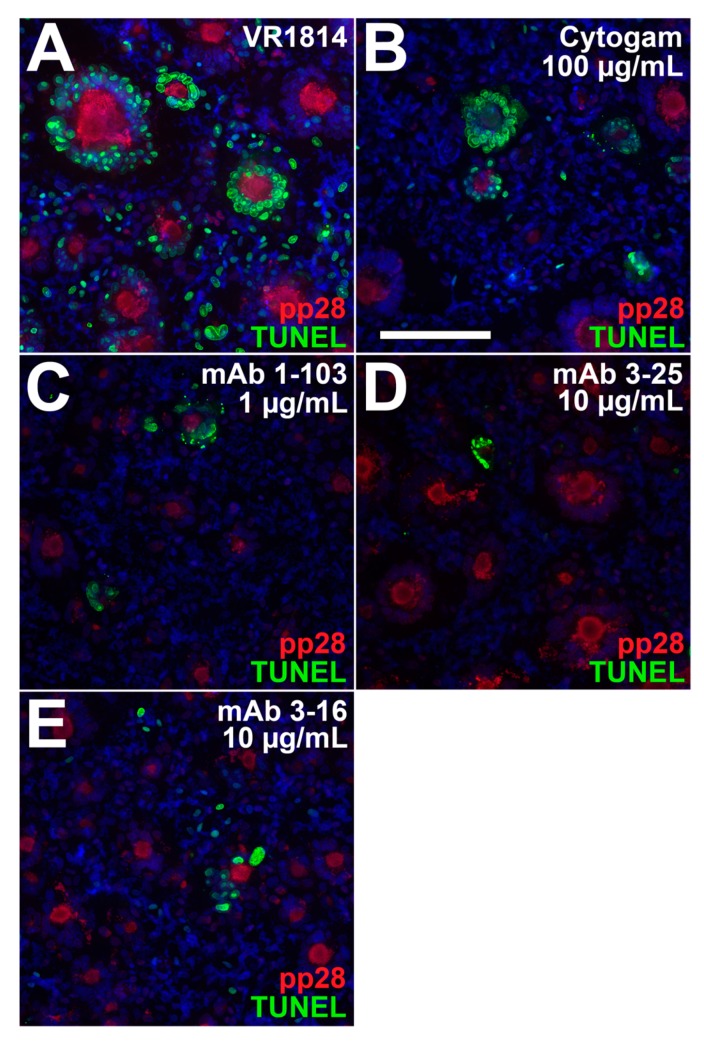
Antibody treatment postinfection reduces syncytium formation and apoptosis in infected ARPE-19 cells. Immunofluorescence staining of pp28 and TUNEL labeling of ARPE-19 cells infected with VR1814 (2.5 PFU/cell) and treated postinfection with (**A**) medium alone, (**B**) 100 µg/mL Cytogam, (**C**) mAb 1-103 (1 µg/mL), (**D**) mAb 3-25 (10 µg/mL), and (**E**) mAb 3-16 (10 µg/mL). Scale bar, panel B = 100 µm. Nuclei were stained with DAPI.

To determine whether reduced virus spread in HCMV-infected anchoring villi could be modeled with neutralizing antibodies in epithelial cells, we performed postinfection treatment experiments in ARPE-19 cells. VR1814-infected cells were treated with mAbs 1-103, 2-18, 3-16, and 3-25 and Cytogam at 1 dpi. At 7 dpi (i.e., six days in the presence of antibody), fewer infected syncytia formed with antibody treatment, and these were smaller, with fewer nuclei, relative to those in untreated infections, showing large syncytia (Figure 9A). mAb treatment also decreased the total number of syncytia formed. Although Cytogam treatment significantly decreased size of syncytia in a dose-dependent manner at 1 μg/mL, total numbers of syncytia formed were reduced at 10 μg/mL. In mAb-treated ARPE-19 cells, levels of gB and pp28 decreased, with both proteins showing more diffuse cytoplasmic localization and less accumulation in VACs (Figure 9B). In untreated cultures, a large percentage of nuclei in syncytia and bystander cells were positive for TUNEL, indicating apoptosis, whereas antibody treatment reduced the frequency of apoptotic nuclei in both syncytia and bystander cells. mAbs 2-18, 3-25, and 3-16 reduced syncytium formation and apoptosis at 100-fold (mAb 2-18) and 10-fold (mAbs 3-16 and 3-25) lower concentrations than did Cytogam (Figure 10).

**Figure 11:**
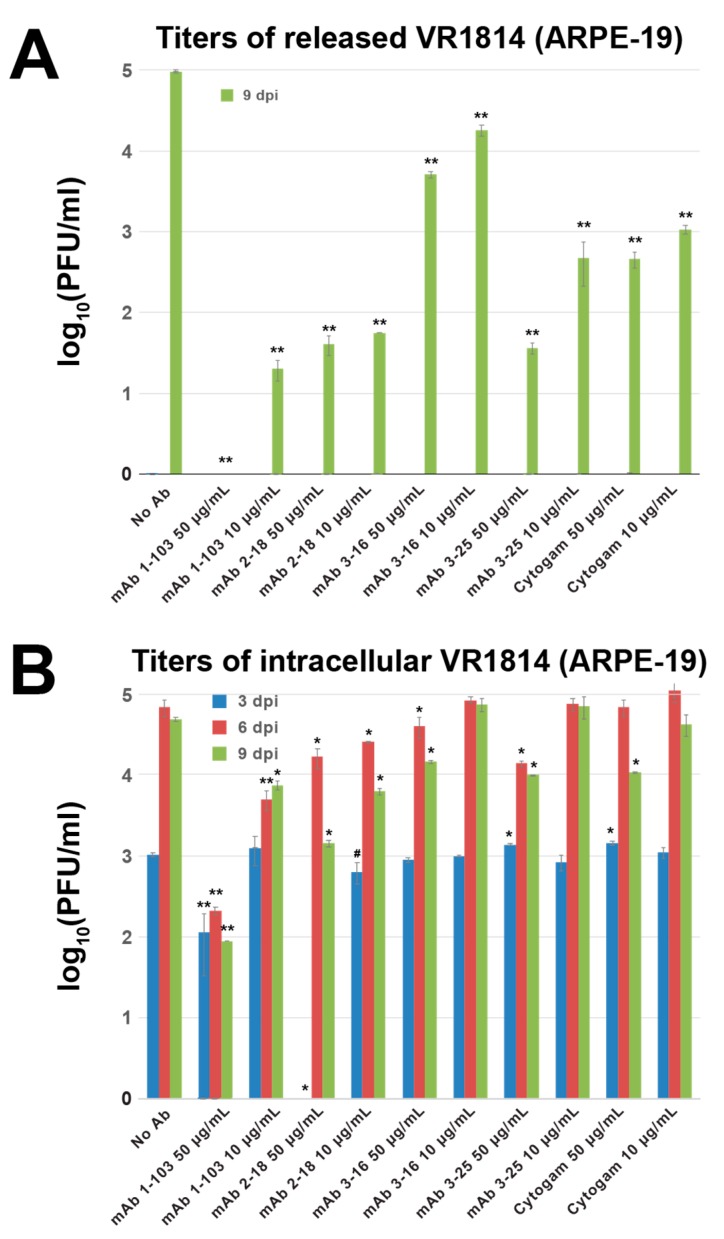
Antibody treatment postinfection suppresses virus release and intracellular accumulation in ARPE-19 cells. (**A**) Representative graphs showing titers (mean ± SE) of released virus and (**B**) intracellular virus at 3, 6, and 9 days postinfection (dpi) in ARPE-19 cells infected with VR1814 and treated with or without antibodies. Significant differences between VR1814 infection alone and with antibodies are shown (^#^ *p* < 0.1, * *p* < 0.05 and ** *p* < 0.01).

Next, we measured intracellular and released virus titers to determine whether reduced syncytium formation with antibody treatment correlated with reduced virus titers. In untreated infected cells, release of infectious virus titers sharply increased at 6 and 9 dpi (Figure 11A). In contrast, cells treated with mAbs and Cytogam did not release infectious virus at 3 and 6 dpi and strongly suppressed virus release at 9 dpi (Figure 11A). In untreated infected cells, intracellular levels of virus increased sharply at 6 and 9 dpi (Figure 11B). Treatment with high concentrations (50 µg/mL) of anti-pentamer mAbs reduced production of intracellular virus and mAb 1-103 continuously suppressed virus production through 9 dpi (Figure 11B). High concentrations of mAbs 3-16 and 3-25 and Cytogam had little effect on intracellular virus at 3 and 6 dpi, and modest effects at 9 dpi. These results show that treatment with neutralizing mAbs reduces syncytium formation, undermines VAC structure, and suppresses virus release in epithelial cells. High concentrations of neutralizing mAbs to pUL128-131 of the pentameric complex and gB somewhat reduce intracellular virus produced.

## 4. Discussion

The specific contributions of humoral and cellular immunity for the prevention of congenital HCMV infection are still unclear. However, it is generally accepted that the presence of maternal antibody before conception provides substantial protection against transmission, whereas primary maternal infection can lead to fetal infection and severe sequelae [54,55,56]. Clinical studies show that frequent high-dose HIG treatment in women who seroconvert in the first trimester significantly reduces transmission, confirming that antibodies play an important role in protection [27], but the antibody specificities and antibody-dependent mechanisms of protection, beyond neutralization, remain to be determined. Recent analysis indicates that antibodies elicited by the HCMV gB vaccine, although moderately protective, had limited neutralizing activity against the autologous virus and no activity toward several heterologous strains in fibroblasts and limited binding to epitopes associated with neutralization, suggesting antiviral activities may have occurred through IgG-dependent Fc receptor-mediated effector functions, including antibody-dependent phagocytosis [57,58,59]. A major limitation in our understanding of the protective properties of antibodies is the absence of specific data on protection within the tissue environment, where cellular phenotypes and states of differentiation can diverge from those of isolated cells. This is particularly important given we have shown that in explants of anchoring villi, HCMV targets cell column CTBs in a differentiation-dependent manner [28,29,60,61,62].

In the present study, we measured neutralizing activities of mAbs specific to HCMV gB, gH/gL, and pUL128-131 of the pentameric complex produced by B-cell clones isolated from healthy seropositive donors in assays to reduce infection in placental cells and explants of anchoring villi from developing human placentas, simulating immunity to HCMV vaccines and antibody treatment following primary infection [34,45,63]. We found that mAbs to gB, gH/gL, and the pentameric complex neutralize infection of primary placental cells and differentiating CTBs at lower concentrations than a commercial HIG product, which has variable efficacy and is not optimized for high titers to any particular epitope [64,65]. The mAbs targeting gB and gH/gL (mAbs 3-25 and 3-16) neutralized HCMV infection in HPFs and TBPCs, whereas mAb 2-18 targeting pUL128-131 of the pentamer neutralized infection in AmEpCs from mid- and late-gestation placentas. Select antibodies showed activity against infection in differentiating CTBs in cell columns of intact anchoring villus explants, with higher neutralizing activity than HIG. Variability in the magnitude of both infection and antibody effects between explants from different placentas reflects variability among individuals. In an analysis of 341 cell columns in 37 anchoring villus explants from four placentas of 8 to 14 weeks’ gestation, mAb 2-18 consistently neutralized infection at a low concentration (0.01 µg/mL), achieving better neutralization at a 100-fold lower concentration than mAbs 3-25 and 3-16, and a 1000-fold lower concentration than HIG. This finding is consistent with a previous report that among the neutralizing monoclonal antibodies elicited by the vaccination of mice, those targeting the pentameric complex were 10- to 1000-fold more potent in neutralizing HCMV infection of primary CTBs from human term placentas than those targeting gH and HIG [20]. At effective concentrations of mAb 2-18, Hofbauer cells in villus cores phagocytosed IgG-virion complexes, preventing infection of CTBs and stromal fibroblasts of anchoring villi. Moreover, mAb treatment after infection reduced virus spread and apoptosis, suggesting that at effective concentrations, neutralizing antibodies could limit virus dissemination and inflammation in developing placentas. Likewise, all mAbs reduced titers of virus released from infected ARPE-19 cells, thereby limiting the spread of infection, reducing syncytium formation, and apoptosis. As in anchoring villi, pentamer-specific mAbs achieved these effects at lower concentrations. Together our results show that potent HCMV-neutralizing mAbs block infection of diverse placental cell types and virus spread in differentiating CTBs, especially mAbs to pUL128-131 of the pentameric complex and gB, which are the most effective.

Cell column CTBs of developing human placentas are susceptible to viruses disseminated in maternal circulation, consistent with exposure of the placenta to blood during vascular remodeling in early gestation that increases by mid-gestation [29]. HCMV DNAemia was detected in 75.7% of women with serologic evidence of primary infection and in 0.5% of women without evidence of primary infection [66]. Moreover, HCMV DNAemia combined with low avidity IgG correlates with congenital transmission [67,68,69]. In cases of primary infection, IgG seroconversion to the pentamer was consistently detected by four weeks after the onset of infection, paralleled by the appearance of virus-neutralizing antibodies effective in epithelial cells and boosted in reactivated infections [70], whereas delayed development of anti-pentamer antibodies was observed in women who transmitted infection [21]. Importantly, HCMV gB-specific IgG also protects placental cells, confirmed here and in earlier studies showing that the potent neutralizing mAb TRL345, reactive with the AD-2 epitope of gB, reduces infection in CTBs of anchoring villi [71] and TBPCs [43] and restores cell functions, and could enable continued growth of placentas throughout gestation [49].

Our initial study of first-trimester placentas infected with HCMV in utero and explants ex vivo showed that CTBs underlying syncytiotrophoblasts expressed IE1, suggesting virions were transferred to susceptible cells within villi through an undefined mechanism [60]. Subsequently, we discovered that strongly neutralizing maternal antibodies conferred protection, enabling transcytosis of IgG-virion complexes by FcRn in syncytiotrophoblasts and phagocytosis by Hofbauer cells, whereas poorly neutralizing antibodies allowed infection of underlying villus CTBs [33]. Endogenous IgG isolated from placentas of HCMV seropositive immune women both reduced infection of villus CTBs and formed immune complexes transcytosed by FcRn [33,48]. Here, we confirm and extend these results and show that HIG and neutralizing mAb 2-18 with high avidity for the pentamer form virion-immune complexes that could bind FcRs on Hofbauer cells in villus cores. It is likely that the receptor FcRγIIA on the surface of Hofbauer cells cooperates with FcRn, the intracellular receptor that transports IgG at a low pH [72,73], leading to the degradation of immune complexes.

We are the first to report that neutralizing antibodies to HCMV reduce virus spread in differentiating CTBs in cell columns of human placentas postinfection and showed that mAb 2-18 was effective at low concentrations, suggesting the high avidity of this mAb could be important [34]. Although the mechanisms whereby infection is suppressed are unknown, altered composition of VACs may play a role, as suggested by reduced accumulation of pp28. Reduced expression of gB and transition of pUL112-113 to nuclear inclusions also suggests delayed replication kinetics. Likewise, studies in ARPE-19 cells showed that HIG and mAbs reduce virus spread, syncytium formation, and released virus titers. Reduced cell-cell spread using mAbs was also reported in infected ARPE-19 cells [45], and syncytium formation was reduced by anti-pentamer mAbs and convalescent phase sera from primary HCMV infection [74]. Whether the mechanisms that limit virus spread in differentiating CTBs and ARPE-19 cells are similar remains to be determined.

Our recent studies and work from others show abundant immune cells reside in the basal decidua [75,76] and could control HCMV infection in the microenvironment of the pregnant uterus [77] by clearing infected cells that are targets of effector memory CD8+ T cells in seropositive women [78]. In addition, antibodies likely contribute to clearing of infection through binding of immune complexes to FcRs on macrophages. In this regard, early development of HCMV-responsive CD4+ and CD8+ T cells was also important for protection from transplacental transmission in women who seroconverted in early gestation [79].

## 5. Conclusions

We have shown that highly neutralizing mAbs directed to pUL128-131 of the pentamer complex, gB and gH/gL perform significantly better than an HIG preparation in neutralization of HCMV infection in primary placental cells and reduce infection in anchoring villus explants of first trimester placentas. These results suggest that treatment with appropriate combinations of mAbs, especially those to pUL128-131 of the pentameric complex, could be significantly more effective than current treatments with HIG to reduce HCMV transmission and congenital disease in pregnancy. Moreover, our results argue that development of highly neutralizing mAbs to pUL128-131 of the pentamer and gB could be critical to achieving immune protection against transmission by vaccination. While immunization with the gB vaccine can elicit both fibroblast and epithelial cell-specific neutralizing responses, they are unable to induce high-titer epithelial cell-specific neutralizing antibodies [80]. Comparison of maternal blood from seropositive women and matching cord blood from placentas has shown that neutralizing titers and avidities of anti-HCMV IgG are comparable, indicating that antibodies introduced to maternal circulation or elicited by vaccines could also protect the fetus from disseminated infection [42]. The ability of mAb 2-18 to reduce virus spread in cell column CTBs suggests antibodies targeting pentamer components and/or antibodies with very high neutralizing activity may confer an additional type of protection that may be essential to effectively prevent transmission and merits further study. A recent report showed that frequent infusion of HIG could provide superior protection against congenital transmission in pregnant women with primary HCMV infection [27]. Since there is no relevant animal model that recapitulates the placental pathology associated with congenital infection, our data in this study could serve as a basis for further evaluation of these mAbs in the clinic for prevention of maternal-fetal transmission.
